# Improving Patient Understanding and Outcomes in Lung Cancer Using an Animated Patient’s Guide with Visual Formats of Learning

**DOI:** 10.1007/s13187-024-02517-7

**Published:** 2024-10-22

**Authors:** Manish R. Patel, Abbie Begnaud, Shanda H. Blackmon, Arkadiusz Z. Dudek, Naomi Fujioka, Janine C. K. Harewood, Pasi A. Jänne, Shirley Kern, Lacey Running Hawk, Ann M. Rusk, Fatima G. Wilder, Robert Winn, Nancy Torrison, Stephanie Searle

**Affiliations:** 1 Division of Hematology, Oncology, and Transplantation, University of Minnesota Medical School, Minneapolis, MN USA; 2 Division of Pulmonary, Critical Care, Allergy and Sleep Medicine, University of Minnesota, Minneapolis, MN USA; 3 Thoracic Surgery, Baylor College of Medicine Lung Institute, Houston, TX USA; 4 Medical Oncology, Mayo Clinic, Rochester, MN USA; 5 Division of Hematology, Oncology and Transplantation, University of Minnesota, Minneapolis, MN USA; 6 Hematology/Oncology, Lee Health Cancer Institute, Fort Myers, FL USA; 7 Lowe Center for Thoracic Oncology, Department of Medical Oncology, Dana Farber Cancer Institute, Harvard Medical School, Boston, MA USA; 8 North Memorial Health Hospital, Robbinsdale, MN USA; 9 Family Medicine, Standing Rock Lakota, Cuyuna Regional Medical Center, Crosby, MN USA; 10 Division of Pulmonary Medicine, Department of Critical Care Medicine, Mayo Clinic, Phoenix, AZ USA; 11 Division of Thoracic Surgery, Brigham and Women’s Hospital, Boston, MA USA; 12 VCU Massey Comprehensive Cancer Center, Richmond, VA USA; 13 A Breath of Hope Lung Foundation, Wayzata, MN USA; 14 Mechanisms in Medicine Inc, Toronto, Ontario Canada

**Keywords:** Lung cancer, Patient education, Health literacy, Knowledge translation, Visual formats of learning, Shared decisions, Health outcomes

## Abstract

Lung cancer patient education resources that address barriers to health literacy, improve understanding, and demonstrate improved patient outcomes are limited. Our study aim was to evaluate and report on learner knowledge improvement and intent to implement behavior change, and validate the benefits of the *You and Lung Cancer* website and YouTube resources. Our study occurred from November 2017 to December 2023. We evaluated audience reach (visit sessions, unique visitors, country origins, page views) and calculated top views by media type (animations, expert videos, patient videos). We assessed the impact and commitment to change through learner surveys (areas of interest, intention to modify behaviors, and intention to discuss disease management with providers) and tested the knowledge of learners pre- and post-reviewing of website content. Our program reached 794,203 views globally; 467,546 were unique visitors; and 243,124 (51%) were unique visitors from the USA. Of US visitors, 46% identified as lung cancer patients. These were patients in treatment (38%), survivors (8%), family members or caregivers (21%), and healthcare providers (14%) with other audiences unspecified (19%). Three areas of highest learner importance were the animations “Understanding Non-Small Cell Lung Cancer” (180,591), “Staging of Lung Cancer” (144,238), and “Treatment and Management of Small Cell Lung Cancer” (49,244). Our study confirmed areas of importance to lung cancer patients and suggests that visual formats of learning, such as animations, can mitigate health literacy barriers and help improve patient understanding and outcomes. Exporting this format of learning to other cancers has the potential to benefit patients and improve health outcomes.

## Introduction

Lung cancer is the leading cause of cancer death globally, with an estimated 1.8 million deaths per year (18%), followed by colorectal (9.4%), liver (8.3%), stomach (7.7%), and female breast (6.9%) cancers [1]The American Cancer Society’s estimates for lung cancer projected for 2024 are estimated to be 234,580 new cases, of which 116,310 are in men and 118,270 are in women; about 125,070 deaths from lung cancer are estimated to occur in 2024 (65,790 in men and 59,280 in women) [2]. Each year, more people in the US die of lung cancer than of colon, breast, and prostate cancers combined [2]. Approximately 654,620 Americans living today have been diagnosed with lung cancer at some point in their lives [3]. Lung cancer is causally associated with cigarette smoking; however, a substantial number of lung cancer patients have never smoked. In the US, it is estimated that 17,000–26,000 annual deaths from lung cancer are in people who never smoked [4].

Despite the large scope of the problem, knowledge about lung cancer among patients and the general public is limited [5, 6]. Furthermore, increased patient understanding has been shown to significantly improve healthcare outcomes [7]. Approximately one-half of all adults in the United States are unable to understand printed healthcare materials, and approximately 90 million adults have fair to poor literacy skills [8]. An individual’s health literacy is an independent predictor of their health-related quality of life with low health literacy being associated with worse overall health, increased hospitalizations, increased complications that require hospital attention, and an overall increase in healthcare costs [9]. Low health literacy is a major barrier to patients’ understanding of lung cancer and their ability to make informed, shared decisions with providers [10–15].

**Fig. 1:**
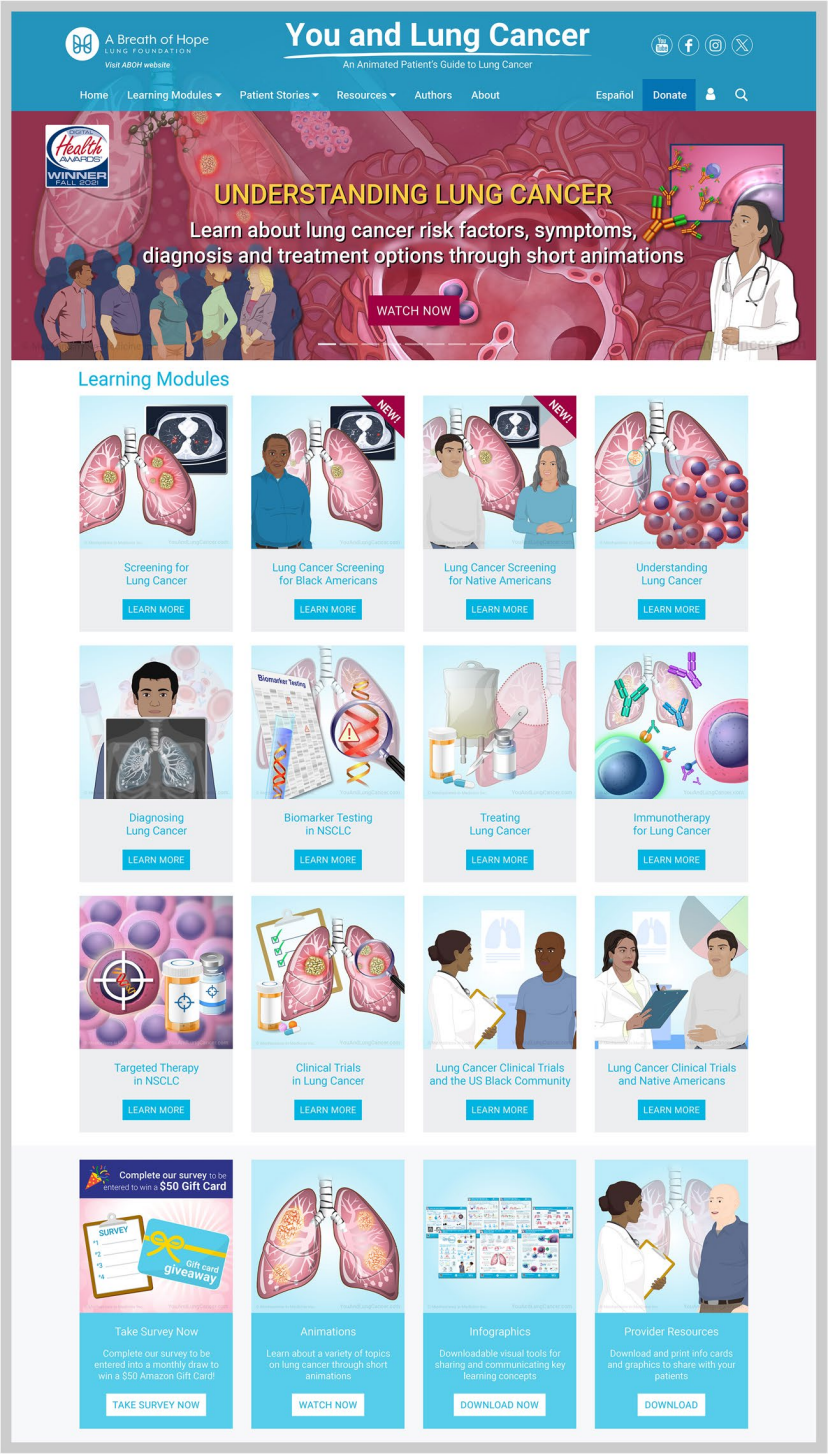
“You and Lung Cancer” website: https://www.YouAndLungCancer.com

To address the problems of limited patient knowledge about lung cancer and the problems of health literacy, *A Breath of Hope Lung Foundation* and *Mechanisms in Medicine* developed the *Animated Patient’s Guide to Lung Cancer – You and Lung Cancer* website (Fig. 1) and YouTube-hosted resources, together comprising a series of educational modules related to lung cancer education, awareness, and understanding, combined with surveys and quizzes designed to measure impact on patient health outcomes. The program was designed to favor audio-narrated animations and visual learning tools, including short (less than 5 min) expert and patient videos, infographics, and picture slideshows, with minimal text-based content. Content is designed and approved by experts in lung cancer, and where necessary, textual descriptions are written to facilitate optimal comprehension, at a sixth-grade or lower reading level, supported by pictures and illustrations [8, 16].

From November 2017 to December 2023, we monitored learner audience use and feedback on this lung cancer patient and caregiver educational resource and evaluated the overall impact of this initiative. The paramount goals predetermining the educational efforts were to (1) improve lung cancer patient understanding; (2) address health literacy barriers; (3) provide educational content tailored to the needs and concerns of patients, families, and caregivers; (4) facilitate informed/shared decisions between learners and their health providers; and (5) improve lung cancer patient outcomes. Here, we analyze retrospective data collected from the educational program over the period of approximately 73 months since its inception to evaluate program usage and impact.

## Materials and Methods

### Study Design and Participants

This is a retrospective study of the *You and Lung Cancer* website and YouTube audience metrics for learner activities related to lung cancer patient education and caregiver lay audiences, conducted from November 1, 2017, to December 31, 2023, a period of approximately 73 months (2252 days in total). At the inception of the *You and Lung Cancer* website launch, a succession of outreach efforts was utilized (YouTube, Google search terms, etc.) to address awareness and facilitate access which included *A Breath of Hope Lung Foundation*’s existing patient and family audiences and de novo audiences in the United States and globally. Although participants visiting the *You and Lung Cancer* modules on the website and YouTube channel are largely comprised of patients, their families, and caregivers, a significant number of users of the program are healthcare professionals.

### Content Development and Access

**Fig. 2:**
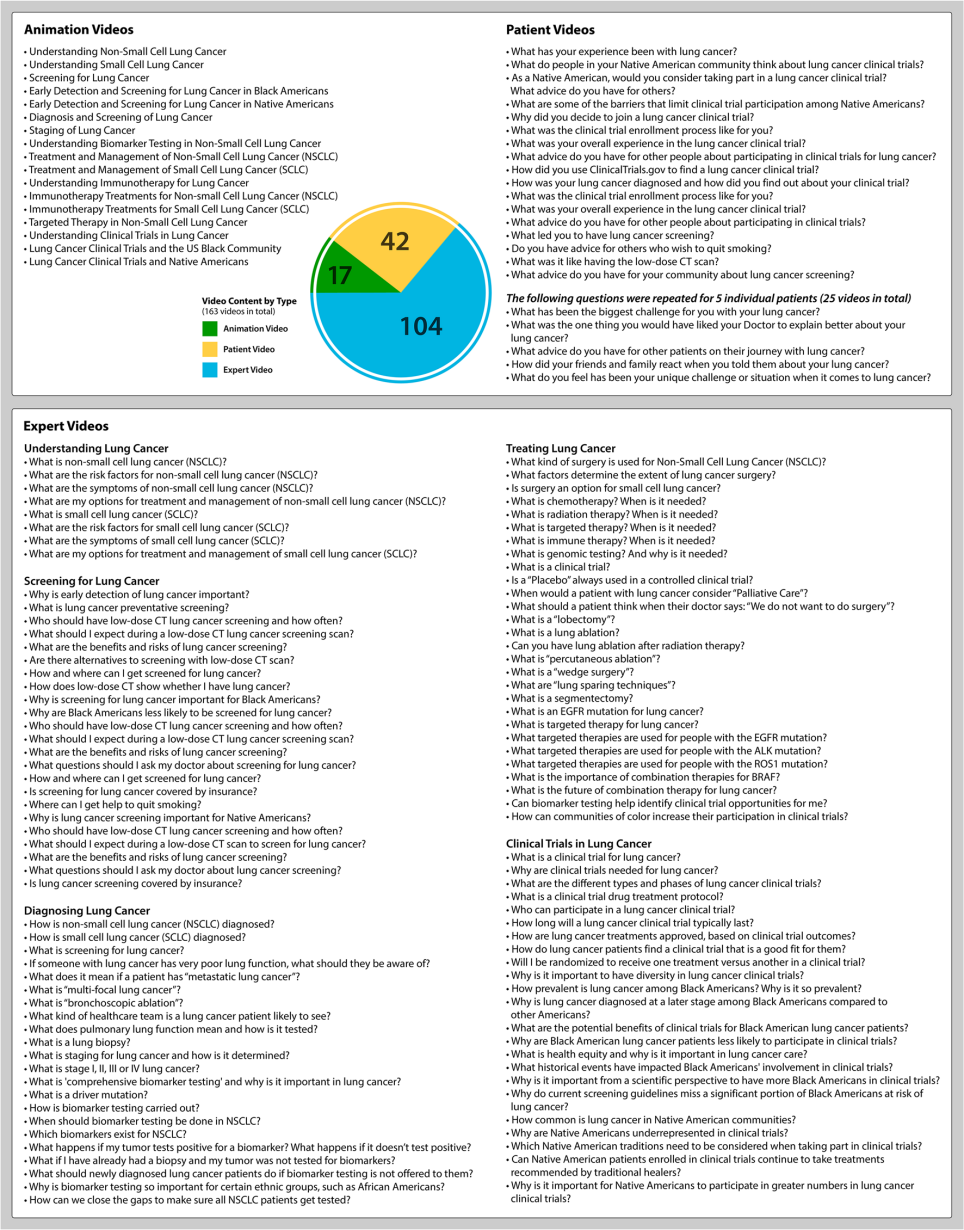
Overview of lung cancer educational program content—animated videos, expert videos, and patient videos from the “You and Lung Cancer” program

The educational content for the *You and Lung Cancer* resources was developed by *A Breath of Hope Lung Foundation*’s multidisciplinary scientific advisors consisting of medical oncologists, surgeons, nurse practitioners, and healthcare providers. Online content is easily accessible, highly visual in nature, and interactive in presentation. The education materials were designed to serve a lay audience with a grade 6 to 8 health literacy level (as assessed by literacy online evaluation tools) whenever possible depending on the medical complexity of the topics. Each module was created to be succinct, practical, informative, evidence-based, and patient-centric and aligned with the chosen learning objectives. The educational curriculum is comprehensive in scope with 163 videos (17 animated videos, 104 expert videos, and 42 patient story videos) covering a wide range of topics summarized in Fig. 2.

Each of the *You and Lung Cancer* animations and videos was developed to be viewed within a short timeframe believed to be conducive to learner retention (ranging from 30 s to 6 min). The videos for the educational modules can be accessed via *A Breath of Hope Lung Foundation*’s website at https://abreathofhope.org/lung-cancer-patient-education/ or directly through the complete online resource, www.youandlungcancer.com, and a supporting YouTube channel at https://www.youtube.com/YouAndLungCancer. The website provides learner interactivity via a learner “pop quiz” (allows users to self-test their knowledge skills), and homepage invitation prompts to provide feedback via optional learner surveys. Pop quiz data was not analyzed as part of this evaluation.

### Learner Surveys

The learner surveys consist of 12 items asking about participant demographics, participants’ experience with lung cancer, participants’ lung cancer knowledge before and after using the website, topic areas of learning resulting from website usage, and anticipated behavior related to website usage. Items on self-reported gains in learning, improved competence, and intent to change behaviors are adapted from Moore et al. [17] and are consistent with level 4 outcomes for continuing medical education. Items assessing lung cancer knowledge pre- and post-website usage utilize 3-point Likert scales (1 = “I didn’t know very much at all about lung cancer,” 2 = “I knew a little about lung cancer,” 3 = “I knew a lot about lung cancer”).

### User Metrics Measurement and Statistical Analysis

Website usage data are reported as frequencies, proportions, and mean (± standard deviation), with 95% confidence intervals where appropriate. We evaluated audience reach, demographics, and metrics such as the number of visit sessions, number of unique visitors based on unique IP addresses, page views, duration of page views, and duration of video views for the *You and Lung Cancer* website and the *You and Lung Cancer* YouTube channel. We also calculated top views, top views by video type (animation, expert video, patient experience video), and top retention values for all videos. Moreover, using the surveys, we assessed the educational impact of the *You and Lung Cancer* program on respondents, including patients and caregivers. We report self-reported learning in the topic areas of lung cancer general information, diagnosis and staging, and management and treatment options. We additionally report participants’ self-reported lung cancer knowledge before and after using the website and utilize a test of marginal homogeneity to determine whether knowledge significantly increased after website use. A test of marginal homogeneity is an appropriate test given the ordinal, paired nature of the data.

## Results

### Participant Characteristics

During the study period of 73 months, the *You and Lung Cancer* website (Fig. 1) for English speakers and YouTube channel had 794,203 total views (*You and Lung Cancer* website = 133,999; YouTube = 660,204). Overall, the educational content was accessed by 467,546 unique visitors from 164 countries. About half of the unique visitors to the website were from the United States (51%), while 49% of unique visitors were from other countries, namely China (8%), Canada (5%), India (4%), the UK (3%), and other countries (29%). The content areas covered in the lung cancer educational program were broad in scope and are summarized in Fig. 2.

Of the 1443 respondents who completed the demographic survey questions, approximately 46% of participant responses identified as lung cancer patients, of which 38% were undergoing treatment and 8% were lung cancer survivors; the remainder were family or caregivers (21%), healthcare providers (14%), and “other” (undefined) (19%) (Table 1). A selected sample list of comments received from patients, family members, caregivers, visitors, and healthcare providers in Table 5 provides verbatim insights into specific areas of importance among the user audience. Some of the patient responses reflected a diversity of interest: comments were received on personal use of immunotherapy, familial predisposition to lung cancer, symptoms related to lung cancer progression, survivorship and unproductive cough, and issues of back pain. Family members commented on outcomes and immunotherapy treatment and concerns about family members who smoke. Healthcare providers commented on the ease of understanding the educational content, intent to share resources with their patients, interest in further resources for patient communities, and requests for resources enabling community dissemination. Unknown comments addressed issues such as back pain; concerns about late-stage detection of small cell lung cancer; and the benefits of access to education materials that provide clear, comprehensive, and informative lung cancer resources.

**Table 1:** Online survey respondent demographics from the “You and Lung Cancer” program

Survey respondent identifies as a:	Count	Percentage
Family member or caregiver for a patient with lung cancer	303	21
Healthcare provider	202	14
Lung cancer patient undergoing treatment	548	38
Lung cancer survivor	116	8
None of the above	274	19
Total	1443	100

### Comparison of Animations, Expert Videos, and Patient Videos

Table 2 lists the most popular topics for the animations. “Understanding Non-Small Cell Lung Cancer” (180,591 views), “Staging of Lung Cancer” (144,238 views), “Treatment and Management of Small Cell Lung Cancer” (49,244 views), “Treatment and Management of Non-Small Cell Lung Cancer” (43,788 views), and “Immunotherapy Treatments for Non-Small Cell Lung Cancer” (42,208 views) were the top animations viewed, respectively.

**Table 2:** Top 5 animations and top 10 expert videos by popularity from the “You and Lung Cancer” program

**Video type**	**Video title**	**APG**^a^	**YouTube**	**Total**
Animations	Understanding Non-Small Cell Lung Cancer	8388	172,203	180,591
	Staging of Lung Cancer	422	143,816	144,238
	Treatment and Management of Small Cell Lung Cancer (SCLC)	554	48,690	49,244
	Treatment and Management of Non-Small Cell Lung Cancer (NSCLC)	2638	41,150	43,788
	Immunotherapy Treatments for Non-Small Cell Lung Cancer (NSCLC)	317	41,891	42,208
Expert videos	What does it mean if a patient has “metastatic lung cancer”?	202	13,273	13,475
	When would a patient with lung cancer consider palliative care?	157	10,666	10,823
	What is a lung biopsy?	498	9449	9947
	What is an EGFR mutation for lung cancer?	159	6708	6867
	How does low-dose CT show whether I have lung cancer?	46	5644	5690
	What are the symptoms of small cell lung cancer (SCLC)?	370	3794	4164
	What is a wedge surgery?	273	2699	2972
	What is non-small cell lung cancer (NSCLC)?	1205	1126	2331
	What are the symptoms of non-small cell lung cancer (NSCLC)?	808	1258	2066
	How is biomarker testing carried out?	107	1466	1573

^a^APG: Animated Patient’s Guide website

The most popular expert videos viewed by the participants were “What does it mean if a patient has ‘metastatic lung cancer’?” (13,475 views), “When would a patient with lung cancer consider ‘Palliative Care’?” (10,823 views), “What is a lung biopsy?” (9947 views), “What is an EGFR mutation for lung cancer?” (6867 views), and “How does low-dose CT show whether I have lung cancer?” (5690 views) (Table 2).

### Viewer Retention for Animations, Expert Videos, and Patient Videos

Table 3 lists the top ten animations and top ten expert videos by viewer retention (as determined by the percentage of the total video duration). On average, 61.25% ± 2.07% (95% CI) of all video content was viewed. Table 4 provides a comparative analysis of all media types (animation videos, expert videos, patient videos) and shows that animation videos have a significantly higher mean number of unique views per day, compared to the expert videos and the patient videos. Although the animations outperformed the expert videos and patient videos in terms of the number of unique viewers, the viewer retention for patient story videos was higher than that for expert videos and animation videos (Table 4). Patient videos had the lowest number of views, but the highest rates of viewer retention with 64.06% ± 4.64% (95% CI) of a patient video watched, compared to 62.74% ± 2.21 (95% CI) for the expert videos and 45.19% ± 3.94 (95% CI) for the animation videos. Table 5 provides a range of feedback via direct comments on the program submitted via the website. Much of the feedback was positive in nature. While we do not have a mechanism to respond to questions directly to patients, the questions received are being tracked and used to incorporate into future modules.

**Table 3:** Top 10 animations, top 10 expert videos, and top 10 patient videos by highest retention determined by the percentage of the total video length viewed from the ‘You and Lung Cancer’ program

Video Type	Video Title	Average Percentage Viewed (%)
Animation Videos		
	Understanding Immunotherapy for Lung Cancer	55.37
	Immunotherapy Treatments for Small Cell Lung Cancer (SCLC)	55.36
	Staging of Lung Cancer	53.70
	Understanding Small Cell Lung Cancer	53.03
	Diagnosis and Screening of Lung Cancer	52.38
	Treatment and Management of Small Cell Lung Cancer (SCLC)	52.1
	Immunotherapy Treatments for Non-Small Cell Lung Cancer (NSCLC)	50.15
	Understanding Biomarker Testing in Non-Small Cell Lung Cancer	49.52
	Understanding Non-Small Cell Lung Cancer	47.68
	Targeted Therapy in Non-Small Cell Lung Cancer	44.05
	Retention for All Animated Videos (*N* = 17)	45.19% ± 3.94 (95% Confidence Interval)
Expert Videos		
	Why do current screening guidelines miss a significant portion of Black Americans at risk of cancer?	98.64
	Will I be randomized to receive one treatment versus another in a clinical trial?	92.79
	What are the risk factors for small cell lung cancer (SCLC)?	82.63
	What should I expect during a low-dose CT lung cancer screening scan?	82.52
	Can biomarker testing help identify clinical trial opportunities for me?	82.01
	Why are Black Americans less likely to be screened for lung cancer?	80.95
	What are my options for treatment and management of small cell lung cancer (SCLC)?	80.20
	What are the symptoms of small cell lung cancer (SCLC)?	79.78
	What is health equity and why is it important in lung cancer care?	79.63
	What are the symptoms of non-small cell lung cancer (NSCLC)?	77.53
	Retention for All Expert Videos (*N* = 104)	62.74% ± 2.21 (95% Confidence Interval)
Patient Videos		
	Mark - What advice do you have for patients on their journey with lung cancer?	87.14
	Julie - What advice do you have for patients on their journey with lung cancer?	86.30
	Mark - What would you have liked your doctor to explain better about your lung cancer?	85.16
	Katherine - What advice do you have for patients on their journey with lung cancer?	83.07
	Curt - What do you feel has been your unique challenge when it comes to lung cancer?	82.61
	Curt - What advice do you have for patients on their journey with lung cancer?	80.93
	Curt - How did your friends and family react to your diagnosis of lung cancer?	80.85
	Julie - What would you have liked your doctor to explain better about your lung cancer?	79.88
	Katherine - How did your friends and family react when you told them about your lung cancer?	79.64
	Curt - What would you have liked your doctor to explain better about your lung cancer?	78.35
	Retention for All Patient Story Videos (*N* = 42)	64.06% ± 4.64 (95% Confidence Interval)

**Table 4:** Comparison of the number of unique views per day for animation videos, expert videos, and patient videos from the “You and Lung Cancer” program

Video type	Mean (views per day)	Standard deviation (95% confidence interval)
Animation video (*N* = 17)	19.22	± 10.34
Expert video (*N* = 104)	0.73	± 0.51
Patient video (*N* = 42)	0.11	± 0.05

**Table 5:** Sample of comments received from patients, caregivers, and visitors from the “You and Lung Cancer” website comment section

Visitor type	Direct comment
Patient	I have lung cancer. I’m looking to use immunotherapy
Patient	I only got my PET scan back this week. My brothers, sister, and 2 nieces have died from lung cancer so I want all the info I can get
Patient	What to expect—symptoms, etc., as lung cancer progresses?
Patient	Breast cancer survivor, unproductive cough. Pain in lungs and lower back. Fever and chills come and go?
Patient	This video is very helpful for first time cancer patients
Patient	I have had an ongoing aching area in one area of my lung that can go through to my back. It has improved since I quit smoking
Patient	Wow, thank you so much for this information which really helped me understand the terminology I was struggling with. Extremely easy to understand. I have been searching for clinical studies related to my cancer and my treatment. I am enrolled in a phase 3 study
Family member	What is the chance to get better if my treatment is immunotherapy?
Family member	Excellent website very informative and easy to understand. Thank you very much for your hard work and helping people worldwide
Family member	Just reading with concern for family members who smoke. Good info to share that may help with prevention of a loved one
Healthcare provider	I appreciate how easy this is to understand. I plan to share this with our patients. Thank you
Healthcare provider	Would love more resources to share with community
Healthcare provider	I am an oncology nurse navigator. Wondering if you have any resources or giveaways I can give to patients and community members?
Unknown	Does it cause pain in the sides and your back?
Unknown	With today’s technology how is it possible for small-cell lung cancer to go unnoticed until patient is in final stages?
Unknown	I found the animation, expert videos, and patient stories all valuable
Unknown	Not a patient, just looking to learn. But great website—content is very clear and comprehensive
Unknown	Thank you very much for making this educational and informative website

### Participant Knowledge Change and Commitment to Change

**Fig. 3:**
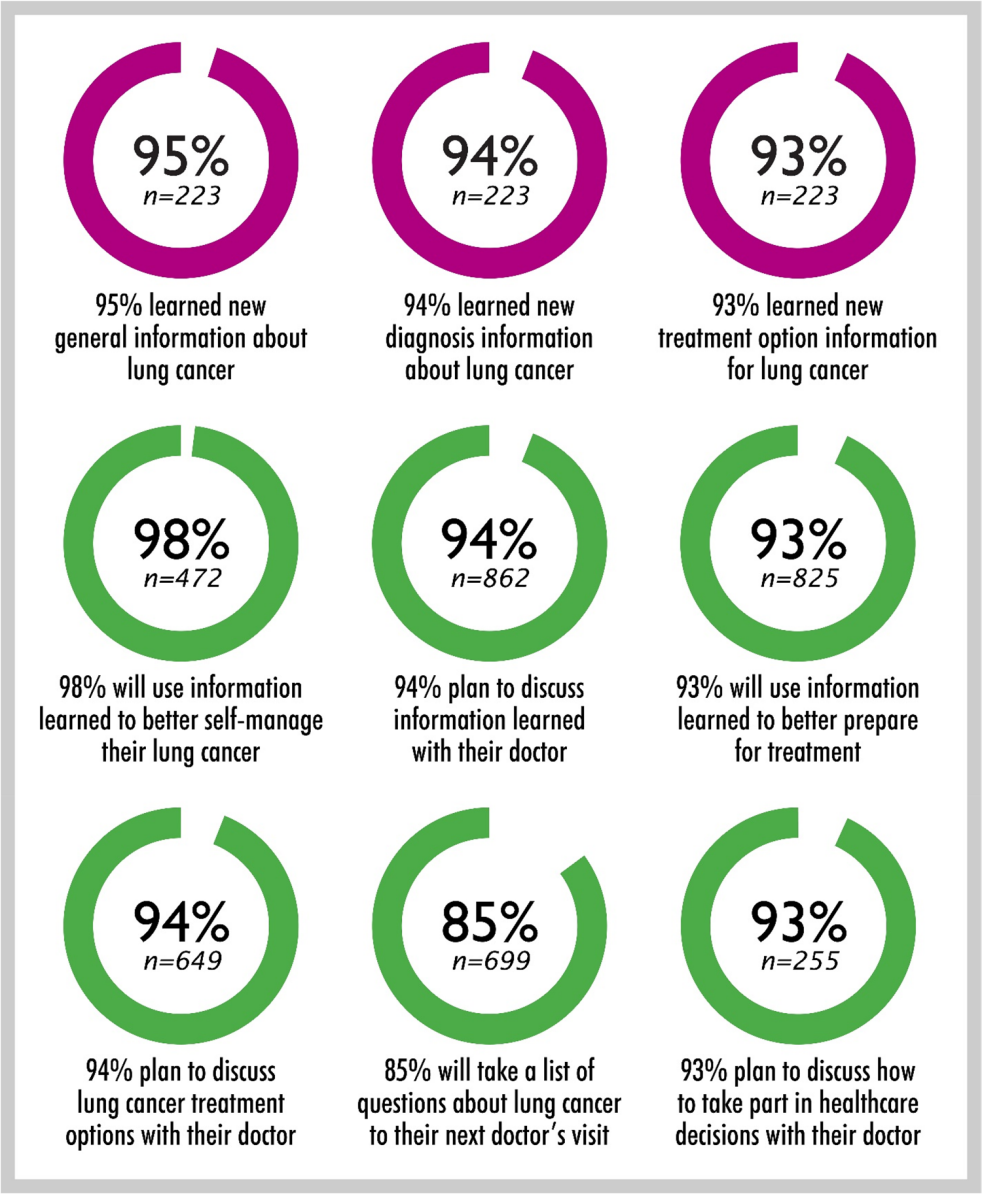
Patients who experienced improved outcomes in the “You and Lung Cancer” program

**Fig. 4:**
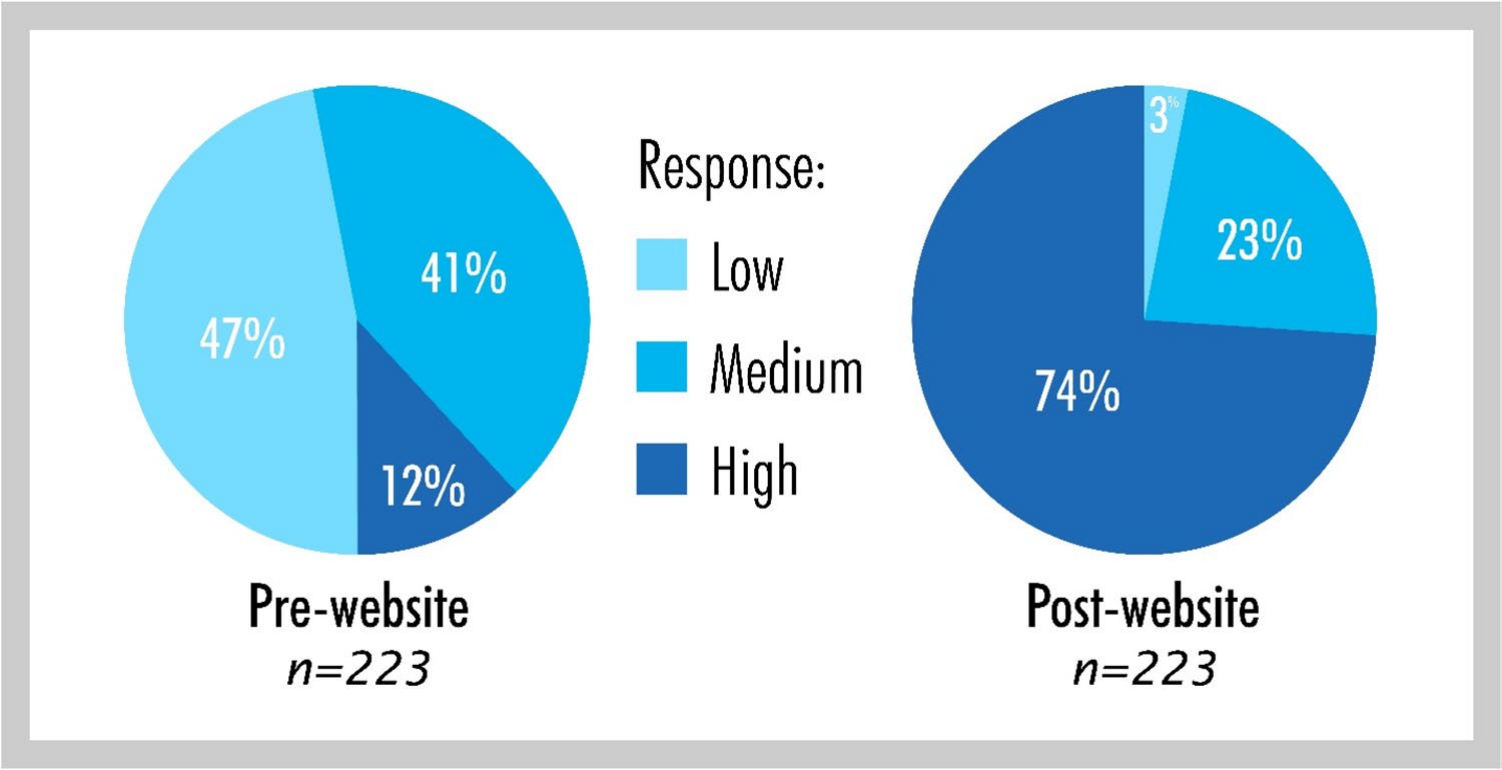
Patients who reported changes in levels of knowledge before using the website and after using the website in the “You and Lung Cancer” program

We report on patients who experienced improved outcomes in the *You and Lung Cancer* program based on their voluntary feedback. Of the 223 participants who completed the online survey on knowledge improvement, approximately 95% reported that they learned new general information about lung cancer, 94% learned new diagnosis and staging information, and 93% learned new management and treatment options (Fig. 3). Most participants expressed a commitment to change, such as using the information to better manage their lung cancer (98%) and their intention to engage with their doctor in discussions about lung cancer (94%) (Fig. 3). Participants also indicated their extent of knowledge of lung cancer before using the website (pre-website) and after using the website (post-website) (Fig. 4): 12% reported high knowledge pre-website, whereas 74% reported high knowledge post-website. Conversely, 47% reported low knowledge levels pre-website, and only 3% reported low knowledge levels about lung cancer post-website (Fig. 4). A test of marginal homogeneity revealed that the increase in self-reported knowledge from pre- to post-website is statistically significant (*χ*^2^(2, *N* = 223) = 268, *p* < 0.001), with 73% of the sample reporting higher knowledge after using the program website than before.

## Discussion

The *Animated Patient’s Guide to Lung Cancer – You and Lung Cancer* uses visual formats of learning to address patient needs and barriers to health literacy and encourages the learners to self-assess their knowledge gains and provides easily accessible, easy-to-follow evidence-based resources [16]. Multidisciplinary expert authors have guided educational content development which provides learners with a comprehensive curriculum to improve and expand their understanding and enable the best achievable health outcomes.

During the 73-month study period, the *Animated Patient’s Guide to Lung Cancer – You and Lung Cancer* education modules garnered 794,203 views and approximately 467,546 unique learner participants. Participants who completed the online feedback survey reported knowledge gains and commitment to change by engaging with their physician or implementing a newly learned self-management action. The feedback survey on self-reported gains in learning, improved competence, and intent to change behaviors is adapted from Moore et al. [17] and is consistent with level 4 outcomes for continuing medical education. Level 4 outcomes can be defined as subjective and self-reported competence and intention to change behaviors. According to Moore, this level of outcome indicates the merits of a continuing medical educational program, which we have attempted to emulate. Our survey data confirms that *You and Lung Cancer* educational content has helped learners make informed choices conducive to shared decisions with their healthcare providers [18].

Educational efforts to improve lung cancer outcomes are limited, and there is a great need for reliable health literacy–oriented, visually informative resources specific to patient needs. The success of this education program may be attributed to its focus on visual learning using animations and videos. In contrast, many cancer patient education resources are predominantly text-based [19–27] and seldom facilitate patient and caregiver interactions, or elicit learner feedback. Little is known about the efficacy of text-based formats of learning to satisfy patient audiences. Furthermore, most text-based patient education resources tend to be written at literacy levels above the comprehension level of laypersons, even though the American Medical Association and National Institutes of Health recommend approximate 6th grade to 8th grade target level for patient education materials [28, 29]. Lung cancer incidence and mortality are highest in groups with socioeconomic disadvantage, including educational attainment [30]. Lower health literacy is also strongly associated with lower socioeconomic advantage, and this audience has the greatest need for education based on low health literacy [31].

The strengths of our educational resource include the wide reach of this program (US and global audience) and the use of multidisciplinary expert faculty from lung cancer centers in the development of content and addressing health literacy impediments using visual approaches (animated videos, expert videos, patient videos, infographics, slide shows). Our data suggests that visual education resources are successful in improving understanding related to lung cancer patient needs and accordingly allow patients to better participate in decisions with their healthcare providers [32].

The limitations of this study include its retrospective design and a relatively small percentage of respondent users (~ 0.3%) who took part in the optional feedback surveys on the *You and Lung Cancer* website. Also, our study is reliant on self-reported data, with no opportunity to independently confirm participants’ reports of their disease history and treatment. A further limitation is the potential for learner response bias in the population who elected to complete online self-evaluation and feedback. Feedback response surveys were optional (no compensation was provided). Because of the nature of the feedback surveys, we were limited in gathering in-depth learner data; however, future goals are to improve the quality of information from heterogeneous learner audiences.

In conclusion, the *Animated Patient’s Guide to Lung Cancer – You and Lung Cancer*, which uses visual formats of learning, demonstrates wide global reach, has meaningful potential to address health illiteracy, and improves understanding and enables shared decisions, which benefit lung cancer health outcomes by informing patients, families, and caregivers. Continued efforts should be made to provide measurable, efficacious lung cancer patient education resources that address health literacy barriers using visual learning pathways to increase disease understanding and improve health outcomes, quality of life, and shared decision-making between patients and healthcare teams. Exporting this format of visual learning to other cancers has the potential to benefit many cancer patients and improve their outcomes.
